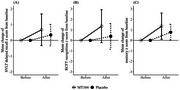# Efficacy and safety of MT104, a dietary supplement based on *Cuscuta* seeds and heat‐killed probiotics, on cognitive function in patients with mild cognitive impairment: A 12‐week, multicenter, randomized, double‐blind, placebo‐controlled clinical trial

**DOI:** 10.1002/alz70859_101851

**Published:** 2025-12-25

**Authors:** Jin San Lee, Hak Young Rhee, Key‐Chung Park

**Affiliations:** ^1^ Kyung Hee University Hospital, Seoul, Seoul Korea, Republic of (South); ^2^ Kyung Hee University Hospital at Gangdong, Seoul Korea, Republic of (South)

## Abstract

**Background:**

Mild cognitive impairment (MCI) represents the symptomatic pre‐dementia stage of Alzheimer’s disease (AD). Given the increasing prevalence of AD and its socioeconomic burden, delaying the progression of MCI to AD is critical. Modulating the microbiota‐gut‐brain (MGB) axis has emerged as a promising therapeutic approach. This study aimed to evaluate the efficacy and safety of MT104, a dietary supplement containing *Cuscuta* seeds and heat‐killed probiotics, in regulating the MGB axis in patients with MCI.

**Method:**

This multicenter, randomized, double‐blind, placebo‐controlled study involved participants randomly assigned in a 1:1 ratio to receive MT104 or placebo. Cognitive function was assessed using the Korean‐Montreal Cognitive Assessment (K‐MoCA) and Korean‐Mini Mental State Examination at baseline and after 12 weeks of treatment. Visuospatial and memory functions were evaluated using the Rey Complex Figure Test and Seoul Verbal Learning Test (SVLT). Statistical analyses included t‐tests, Mann–Whitney U tests, analysis of covariance (ANCOVA), and ranked ANCOVA.

**Result:**

The mean changes in verbal memory function, as measured by SVLT delayed recall, showed clinically significant improvement in the MT104 group compared with the placebo group in the intention‐to‐treat and per‐protocol groups. The global cognition, as measured by the K‐MoCA, also significantly improved in the per‐protocol group. Additionally, there were no significant findings regarding the safety profile of MT104.

**Conclusion:**

MT104 improved memory performance and global cognition in patients with MCI without safety concerns. These findings support the potential of dietary therapeutic strategies to reduce the risk of progression from MCI to AD dementia. Further studies are needed to confirm these results and explore the long‐term benefits of MT104.